# The Hospital Nursing Supplement

**Published:** 1895-03-23

**Authors:** 


### "The Hospital" Nursing Mirror.

Being the Extra Nursing Supplement of "The Hospital" Newspaper.

[Contributions for this Supplement should be addressed to the Editor, The Hospital, 428, Strand, London, W.C., and should have the word "Nursing" plainly written in left-hand top corner of the envelope.]

### News from the Nursing World.

### A VISIT TO ST. MARY'S HOSPITAL.

His Royal Highness the Duke of York spent an hour and a half in the wards of St. Mary's Hospital, of which he is president, on Friday, 15th inst. The Duke was accompanied by the Duchess of York, and the senior physician of the hospital, Sir William Broadbent, conducted their Royal Highnesses through the institution. Much pleasure was given to the patients by the distinguished visitors speaking in turn to each of them. The Duchess also handed a bunch of violets to every sick person, and their Royal Highnesses both expressed much interest in all that they saw in the hospital.

### ROYALTY AT ALDERSHOT.

After the Empress Frederick had inspected the Army Gymnasium at Aldershot last week she went over the Cambridge Military Hospital. The Empress was accompanied by H.R.H. the Duchess of Connaught.

### EAST-END MOTHERS' HOME.

The annual meeting of the East-end Mothers' Home was held at Lowther Lodge on the 18th inst., by kind permission of the Hon. William and Mrs. Lowther. H.R.H. the Duchess of Albany was present with a number of other guests. Sir Michael Hicks-Beach presided, and spoke of the claims of this charity, established in the very midst of the people who needed its help. Mrs. Temple moved the adoption of the report, and spoke of the interest taken in the work by herself and the Bishop of London. The success of the East-end Mothers' Home appeared to be acknowledged by all present, and it is to be hoped that the sympathy shown may take a substantial form, and help to remove the debt of £1,050 which remains on the building.

### LONDON HOSPITAL PROBATIONERS.

The services of Miss Stirling Hamilton have been secured by the London Hospital Committee to carry out the new scheme for granting preliminary instruction to pupil probationers. The work done by Miss Stirling Hamilton during the last three years at the Government Hospital, Gibraltar, has been as excellent as that which she did previously as probationer, nurse and night sister at the London Hospital, and many old friends and fellow-workers will be glad to hear of her appointment at Tredegar House, where, as noted in The Hospital of last week, the new educational scheme will be carried out. The certificate of training is given at the end of two years at the London Hospital, and from those regular probationers who gain it a third year of service is required either on the private or hospital staff, according to vacancies, individual fitness, or health. It will thus be seen that every probationer is tied to the hospital for three years by her engagement, although only two years of that time may be spent in actual training. Doubtless the new departure in regard to preliminary instruction will share the success which has attended the many other excellent schemes by which Miss Lückes has now for many years markedly advanced the cause of trained nurses.

### TRAINING AT LAMBETH INFIRMARY.

The Lambeth Infirmary Committee have conferred with Dr. Downes, the Local Government Board Inspector, regarding the proposed abolition of the office of "Superintendent of Nurses and Assistant Matron," created some two years back. Dr. Downes explained that the training of probationers and the classes of instruction supplementary to the lectures of the medical officer, had proved most satisfactory in other poor law infirmaries, and that no such difficulties had arisen elsewhere as had been found at Lambeth. The committee have therefore decided to advise the Guardians to give the scheme another trial, and to rescind their resolution to abolish the office. It was further recommended that there should be explicit rules defining the duties; that the salary should commence at £40, rising to £50 per annum; and that the Superintendent should be subordinate to the Matron. The resignation of Miss Tilbury, which we noted on March 2nd, will therefore be followed by the appointment of a successor under somewhat changed conditions. The nurses and probationers have presented a handsome dressing-case to Miss Tilbury, accompanied by a letter expressing their gratitude for the excellent instruction which she has given them.

### PROBATIONERS IN FEVER HOSPITALS.

The Metropolitan Asylums Board had recently under consideration a report from the Nursing Staff Committee, dealing with the training of probationers in the Board's fever hospitals. It was asserted that at present a large proportion of the nurses remained less than a year, and it was thought that by the introduction into the hospitals of probationers for a stated term of training a supply of better qualified fever nurses would be secured. An amendment proposed and, after some discussion, carried, was, "That overtures be made to the nursing schools with a view to completing the three years' course by some training in fever nursing, after the foundation of a good nursing education has been laid in a general hospital or infirmary."

### THE NORTH LONDON NURSING ASSOCIATION.

The annual meeting of this association took place last month at the Home, Mr. H. C. Stephens, M.P., being in the chair. A sale of work in aid of the funds is to take place in May, increased financial support being urgently needed by the association. Some difficulty has been experienced of late in obtaining nurses, and letters for convalescent homes are also in request. Some of the Great Northern Hospital's out-patients are visited by the North London Nursing Association by request of the medical staff.

### THE BIRMINGHAM NEEDLEWORK GUILD.

During its twelve years existence the Birmingham Needlework Guild has supplied 273,269 garments to poor people; 36,823 of these having been sent in this winter. At a well-attended meeting held on the 4th inst., under the presidency of the Viscountess Newport, many representatives of charitable associations testified to the value of the work of the Guild, which has ninety-nine branches in the Birmingham centre. Fifty-one of these are connected with the Church of England, and the others to various religious denominations. Amongst the institutions which have benefited, the Birmingham General Hospital was named as the recipient of 500 garments. The St. Vincent de Paul Society, the Society for Giving Country Holidays to Town Children, the District Nursing Society, the Jewish Charities, the Emigration and the Charity Organisation Societies have also received handsome consignments of clothes. The Birmingham Needlework Guild originated by Mrs. H. W. Edmunds, is desirous of obtaining more members and of organising additional branches. The success already achieved in Birmingham ought to lead to the establishment of similar associations in every district in England.

### NOT "A WASHER."

"Did you have the nurse to look after you?" asked the squire's wife of a poor woman who had been descanting upon her recent illness. "Well, ma'am, I was just wantin' a chance to ask you about her," responded the convalescent, "if you'll excuse the liberty. We all wants partickler to know if that ere person is rightly a nurse at all? She ain't a washer, that's certain." The squire's wife hastily replied, "Oh, Mrs. Brown, you can't expect her to do the family washing; she wouldn't have time to attend on the sick in the village and do that too." Mrs. Brown flushed hotly. "I should just say not, and we don't want her to. Any neighbour can do our bits o' clothes as well, or p'raps better'n any nurse. What we can't understand is clean folks like you and the squire wishing for us to be content with a ignorant woman as never changes a sick woman's bed, nor gives her a reg'lar good all-over wash! Of course, people says as you fancies cheap nurses quite good enough for the likes o' we. Naturally you didn't know," added the woman in a more conciliatory tone, "as most folks spots a proper nurse directly. That's the sort as we calls 'washers,' cos they've been learnt to make sick people nice, and teach 'em the right way to do things. Some of us wanted to tell squire and you that it's folly to chuck away money over this cheap sort. We'd as lieve be without them if we wasn't unwillin' to seem ungrateful!"

### NURSES AS ALMONERS.

In the report of a nursing association recently brought to our notice, mention was made of monetary assistance which had been given to certain patients. Most people having a practical knowledge of the nursing of the sick poor in their own homes protest against a system which converts a nurse into an almoner. A nourishment fund should certainly be allied to every district hospital or nursing centre, in order that butter, milk, eggs, &c., may supplement the work of the nurse, when poverty and sickness are co-existent. The actual distribution, however, is better left to others, the ladies who supply the diets being responsible for their punctual delivery. The giving of money by the district nurse to the sick whom she visits must imperil her position with patients who without such bribes should appreciate her valuable services. These she gives alike to the thrifty and the destitute, but almsgiving must arouse distinctions which are beyond her province, and likely to embarras her work.

### CORDIAL CO-OPERATION.

The local medical men and the clergy of all denominations recently signed a requisition to the chairman of the Town Commissioners at Dundalk, which resulted in a public meeting being called to discuss the formation of a district nursing society. In Dundalk the success of an association for providing skilled nurses for the sick poor in their own homes is certainly assured by the cordial and broad-minded support given to the movement. The new association will be affiliated with the Queen's Jubilee Institute.

### HINCKLEY COTTAGE HOSPITAL.

The past year has been made memorable to Hinckley by much sickness, both smallpox and diphtheria having been unfortunately prevalent. The services of the parish nurse have been much valued and in constant demand. The strain on the public charity made by the epidemics has resulted in reduced subscriptions to the Cottage Hospital and to the Parish Nurse Fund. This decrease will no doubt be rectified, for both charities are well managed, and thoroughly appreciated. Twenty-five persons came into the hospital for operation, sixty-five being the total of in-patients nursed in the year. The committee congratulate themselves on having retained the valuable services of Miss Rodgers as matron, and they also speak with appreciation of the parish nurse and probationer. In the district, 6,391 visits have been paid to 450 patients, including 193 cases of diphtheria.

### HINDU BATHERS.

The origin of many an illness is the cold caught at a funeral, and we are apt to put this down to our lamentable climate; but an Indian correspondent shows us that, much as the graveside is a danger in the variable weather of England, so even in India funeral rites are attended with parallel risks. The Hindus always bathe in a river after assisting to burn the dead, and in some seasons the weather renders the practice a dangerous one. It is reported, in fact, that after every Hindu funeral two or three of the mourners are stricken with pneumonia.

### VENEZUELAN RED CROSS SOCIETY.

The President of the Republic of Venezuela consented to become Honorary President of the new branch of the Red Cross Society inaugurated last month at Caracas, and the Archbishop of Venezuela has been made Vice-President. The work of the society, outlined at the Geneva Convention, consists in the establishment of ambulances and hospitals in time of war; in preparing for the exigencies of war in times of peace; and in providing courses of instruction in First Aid to the injured. So much enthusiasm has been shown in the formation of this branch of the Red Cross Society that there is every prospect of its becoming a most influential one.

### SHORT ITEMS.

The Garston District Nursing Association, affiliated with the Queen's Jubilee Institute, has laid a satisfactory report of the year's work before the subscribers. —A performance in aid of the funds of the Red Cross Society took place at Nice on 18th inst. H.R.H. Prince Louis of Battenberg was amongst the visitors present, and the entertainment, which was very well attended, was held in the Casino Municipal.—It is proposed to have a permanent isolation hospital at Hinckley.

### Royal British Nurses' Association.

The monthly meeting of the Registration Board will take place at the offices of the association, 17, Old Cavendish Street, at five p.m. on Friday, March 22nd.

The quarterly meeting of the General Council is fixed for April 19th, at five p.m., at the same address.

The Modern-Trained Nurse.

A lecture on "The Modern-Trained Nurse" was given by Sir Dyce Duckworth, M.D., at 17, Old Cavendish Street, on March 15th. The chair was taken by Mr. Herbert Page, F.R.C.S., and there was a good attendance of nurses.

Sir Dyce Duckworth said, when asked to lecture to the members of the association, it seemed to him that he might do worse than give the ideas concerning "The Modern-Trained Nurse," which had been ruminating in his mind for some time past. One special feature in the history of the nineteenth century would be the systematic training of women for the care of the sick. Florence Nightingale's memory and labours would ever stand out amongst its glories, and the English nation might be proud of the development of the nursing profession; no other country could show anything like it, save America. Germany was far behind, and nursing in France was a disgrace to a civilised country; the same might be said of other Continental countries. It was not to be expected that changes had come about without friction, but for the most part the system which produced the modern-trained nurse had been welcome. There were differences of opinion among authorities as to the exact length of time requisite for the efficient training of a thorough nurse. The British Nurses' Association had determined that no woman could be sufficiently trained in less than three years, and thought it necessary to add to practical duties systematic instruction in physiology; but many nurses could not afford to set aside four years of life for training, and on many sides were to be heard complaints of the cost of nurses. Not that trained, registered nurses, were overpaid—the case was frequently the opposite—but the limit of remuneration was as high as could be borne, and many people could not pay even half the ordinary charges. The public was its own master in this matter, and would have nurses they could afford to pay for. The question was, treating the whole subject from a broad point of view, could any other class of nurse be recognised in the association; could a lower grade be encouraged? What was to be done with the many nurses who would never come up to the highest standard? A vast amount of good work was being carried out by persons whom the association considered insufficiently trained. People said that as much instruction could be given in two years in some institutions as in three at others, and he (Sir Dyce) would not deny this. There were many institutions in London where two years' training was the rule. Sir Dyce himself had long thought it possible to establish two or three grades of nurses, with varying claims for remuneration, but this could only be carried out under State registration. No help could be looked for from the public, to whom one nurse was as good as another. Certain charges made against highly-trained nurses were regarded by the lecturer as very serious. It was said that they were apt to take up spheres of duty which did not belong to them, so that some medical men preferred to entrust their patients to less highly-trained women; but he asserted that such conduct could only arise from insufficient training. With regard to the comparative value of training in London or country hospitals, he gave the preference to the larger hospitals. He urged all nurses, both during and after their training, to respect and venerate their matrons.

Sir Dyce caused much amusement to his audience by a hearty recommendation of matrimony in the consideration of their ultimate future. He thought a good nurse should make a "model wife and a happy mother," and took strong exception to the "modern cant and nonsense which decried marriage."

Mrs. Spencer, hon. secretary of the association, commented on the length of training considered desirable for nurses, saying that some hospitals increased the period of training to four years, which she considered much too long. If a woman of twenty-three trained for four years, she would then be twenty-seven, and at thirty-five it was often said that she was too old for nursing. Two years in a general hospital should be sufficient, and Mrs. Spencer approved of a third year in a special hospital, particularly for those taking up private nursing.

Miss Isla Stewart strongly supported the four years' training, which was, she believed, only carried out in London at St. Bartholomew's and the Royal Free Hospital.

Mr. Page spoke in favour of three years' training rather than two, and Sir Dyce Duckworth agreed with him.

With a vote of thanks to lecturer and chairman the meeting broke up.

### Lewisham Infirmary.

At a meeting of the Lewisham Board of Guardians on Monday, the chief interest centred in a further discussion with regard to the infirmary matters. A report of the Infirmary Committee was laid before the Board, recommending that the Matron, Miss Patteson, should be requested to "revoke in writing her refusal to obey the medical superintendent's orders," and to give an undertaking that she would in future carry them out.

The Chairman, Canon Bristowe, did not consider it fair now to ask the Matron to withdraw words spoken under very different, and very strained circumstances. He believed she was quite ready and willing to go on with her work, and advocated that she should be merely asked to return to her duties, the Board relying upon her carrying out their instructions in the future.

Mr. Brookes thought it was no good to go on fighting the Local Government Board. As the Matron had to come back it would be better to have her back at once.

Mr. Brunning said the whole matter was a most unfortunate legacy from the old Board. He had come to the conclusion that if there were to be further fighting and the matter honestly and fairly settled it must be thoroughly re-opened.

A great deal of discussion followed, in the course of which it appeared that although it was stated that the members of the infirmary staff had free access to the Board, yet to a "round robin" addressed to the chairman by a number of the nurses no reply had been given, one of the Guardians remarking that a "round robin" was "as bad as an anonymous letter," and it "was not thought fit to bring it before the Board." Mr. Brunning said that as a matter of courtesy the Board's servants had a right to a reply.

The Rev. Dr. Marshall thought that asking the Matron to obey orders "whether right or wrong" amounted to interference with a person's moral sense, which might lead to misunderstandings, whereas some modification of the order might conclude the matter.

A certain amount of light seemed to be thrown upon the position of the Board by a remark of Mr. Kemp's, who, in saying that there had been no action on the Matron's part to modify the state of affairs since the Guardians had decided that for the well being of the Infirmary she must resign, and that therefore they should maintain the attitude then taken, added "What will our medical superintendent think of it?" After further argument it was suggested that Miss Patteson should be called before the Board, the rules under which she had taken office be read over to her, and the chairman shoul l then ask her if she were prepared to abide by them, and carry out instructions to the best of her ability.

Miss Patteson was then called in and the Local Government Board orders were read. The Chairman then said that the Board would like to hear from herself whether, upon being reinstated, she would be prepared to carry out these rules loyally, thoroughly, and heartily, Miss Patteson's reply being, "I will endeavour to do so." The Chairman: "As far as we understand, you are wishful to resume your duties?" Miss Patteson: "Yes." Miss Patteson then asked if she would be at liberty to make any statement to the committee should occasion arise, and was informed that in future all reports entered in the report book would come before the committee.

Miss Patteson was evidently suffering under considerable nervous tension, and unfortunately managed to convey a decidedly unfavourable and probably far from accurate impression, no doubt quite unconsciously, with the regrettable result that those guardians who had previously been in favour of her return to the infirmary without further delay, after the interview, expressed themselves willing to support the resolution of the Infirmary Committee. At this point a letter from Miss Patteson was received by the chairman, which should have been delivered before the commencement of the meeting, and which was as follows:—

March 17th, 1895.

Rev. and Dear Sir,—In reply to yours of the 16th inst., I beg to say that I shall be very pleased if the result of to-morrow's board-meeting is to name an early date for me to resume my duties as Matron. If there is anything that has been mistaken or misunderstood in my conduct towards the Board of Guardians, I would willingly explain it personally to the board, if so desired, as far as it lay in my power to do so. My letter to the Medical Superintendent, dated October 26th, 1894, expresses my views as to the position I believe it is my duty as Matron to hold towards that officer, and I am quite ready to now endorse what I then wrote. My most sincere thanks are due to the guardians, who, at the expense of much time and trouble, have endeavoured by thorough investigation to clear certain matters up relating to my suspension from office. Kindly lay this letter before the board, with the assurance that my only wish is to perform my duties efficiently and quietly without being misrepresented.—Believe me, yours faithfully,

Eleanor L. T. Patteson.

To Rev. Canon Rhodes-Bristowe, chairman of the Lewisham Board of Guardians.

Finally the report of the Infirmary Committee was adopted *nem. con.*

The question of the two assistant matrons then came up, calling forth further differences of opinion, Mr. Wilkinson and Mr. Lay being anxious that they should, before being reinstated, reply to certain questions requesting them to "loyally obey" their immediate superiors, while Mr. Brunning contended that repeated expressions of regret had been received from Miss Campbell and Miss Mills, and that it was "an insult to these intelligent ladies to ask them to carry out their duties 'loyally.'" These two ladies had evidently made a satisfactory impression upon the majority of the guardians, and on the question being put to the vote it was decided by a majority of two that their previous letters of explanation should be accepted.

Miss Patteson's reply to the communication to be made to her by the Board will be received and considered at an adjourned meeting of the whole Board.

### Appointments.

Dewsbury and District Infirmary.—Miss Lilian S. Newman has been appointed Matron of this infirmary. She was trained at the Leeds General Infirmary, was Matron of the Scarborough Hospital, and Superintendent of Nurses at Smedley's Hydropathic Establishment, Matlock. We wish Miss Newman every success.

Lytham Cottage Hospital and Convalescent Home.—Miss Darnbrough, at present Matron of the Berkeley Cottage Hospital, Gloucestershire, has been appointed Matron of the Lytham Cottage Hospital, and takes many good wishes with her to her new work.

### East London Nursing Society.

The Lord Mayor was unfortunately unable to take the chair at the annual meeting of the East London Nursing Society; the Rev. Prebendary Harry Jones (chairman of the committee) therefore presided over the large gathering at the Mansion House. Canon Elwyn, the Rev. J. F. Kitto, the Rev. E. Hoskyns, and the Rev. M. Hare all bore testimony to the excellent work done by the society, and deplored the serious decrease in donations in the past year. There have been special gifts for supporting nurses in new districts, but the financial difficulties of the society make it probable that some of the old districts will have to be given up. This would be most disastrous to the poor, by whom the nurses are highly valued, and all the speakers appeared to regard with consternation the prospect of any such reduction in the number of the workers. During last year 101,641 visits were paid to 4,291 patients, and such a record of work amongst the sick poor of the East-end ought to appeal strongly to many who have the power to dispel by prompt liberality the pecuniary difficulties of this well-known nursing society.

It appears from the report that a most liberal proposal has been made in the course of the year by a gentleman who offered to supply a Central Home in which six or eight nurses might live under the direct superintendence of a matron. Many opinions in favour of such a plan have been expressed by "the highest authorities on nursing." After fully considering the matter the committee of the East London Nursing Society, however, decided that it was advisable to cling to their original scheme of letting each nurse live alone under the supervision of a resident who provides the lodgings, and "an assistant lady who watches her work, helps her with advice, and provides all extra food or relief that may be needed for her patients."

### Presentations.

On leaving Coldstream Cottage Hospital, Nurses Thompson and Brewer were each the recipient of a purse containing five sovereigns, in token of the esteem and respect felt for them in the district.

On the occasion of her resignation of the post of Matron of the City of London Lying-in Hospital, Miss K. M. Heanley was presented by her pupils with an Indian tea service and tray of beautiful workmanship. Miss Heanley proposes to take up lecturing, in which we wish her all success.

Mrs. Sinclair, Matron of Belvidere Fever Hospital and its branch hospital, Kennedy Street, Glasgow, completed the twentieth year of her matronship on Friday, the 15th inst., when she was presented with a number of costly presents. From the medical staff a solid silver tea service elegantly engraved; from the nursing staff of Belvidere a massive gold bracelet studded with pearls, and a magnificent silver and gold cake basket of elaborate workmanship and unique design; from the nursing staff at Kennedy Street a beautifully polished oak tray; from the two assistant matrons and the superintendent of the stores, and her assistants, a very handsome drawing-room clock; from those employés who come immediately under Mrs. Sinclair's supervision, one dozen of handsome silver fruit knives, in satin lined case. The presentation was made by Mr. Hall, chaplain to the hospital, in the presence of Dr. Johnston, the medical superintendent, and a large number of charge nurses and assistant nurses—a band of amiable and devoted women, many of whom are now approaching their second decade in the service of this hospital. In a few well-chosen words Mr. Hall spoke of the many changes for the better that have taken place since Mrs. Sinclair entered upon her duties as matron twenty years ago. How she has seen it grow year by year from a small place to what it now is—a very large town. Mrs. Sinclair, who was visibly affected, addressed her "dear nurses" in terms of affection, thanking them for their beautiful presents and for the noble way that each one performed her duties under many trying difficulties and uphill work in the years that are past, whilst taxing their strength and health to the utmost unselfishly and faithfully for the comfort and well-being of their patients and the prospects of the hospital generally. Mrs. Sinclair also spoke of the thoughtful care, the warm sympathy, and valuable assistance afforded her on many trying occasions by Dr. Allen, late Medical Superintendent of Belvidere.

### The Royal National Pension Fund for Nurses.

The eighth general meeting of members of the Royal National Pension Fund for Nurses was held at the offices, 28, Finsbury Square, E.C., on Thursday, March 14th, 1895. Mr. Walter H. Burns, Chairman of the Council, presided, and there were, amongst others, present: Messrs. Henry C. Burdett, Arthur H. A. Morton, G. Norman, E. C. Perry, M.D., Thos. Bryant, F.R.C.S., Herbert C. Gibbs, Hugh M. Macpherson, George King, F.S.S., &c., Miss Leigh, Miss Wilson (Workhouse Infirmary Nursing Association), Miss Vincent (St. Marylebone Infirmary), Miss Nott-Bower (Guy's Hospital), Miss Pritchard, Miss Gordon (St. Thomas's Hospital), and Miss Gordon (Charing Cross Hospital).

The Secretary (Mr. Louis H. M. Dick) having read the notice convening the meeting,

Messrs. King and Macpherson kindly undertook the duties of scrutineers in connection with the ballot for the election of seven representatives of the annuitants and policy-holders of the Society on the Council.

The report and balance-sheets, which had already been printed and circulated, were taken as read.

### MR. WALTER H. BURNS' ADDRESS.

The Chairman, who was received with a hearty round of applause, said: As usual, you will expect your Chairman to supplement by a few more details the very full report which has been issued by the Council and distributed among the members of the Fund.

But before proceeding with such statement, I desire to record the profound grief which we have all of us felt at the death of our valued colleague, Mr. Clifford Wigram. This gentleman was with us in the very earliest stages of the formation of our Fund, and by his constant attention, great experience, and devoted interest, contributed very much to our successful establishment.

I am glad, however, to be able to add that Mr. Herbert C. Gibbs, a member of the firm of Messrs. Antony Gibbs and Sons, who contributed £5,000 to our original starting fund of £20,000 has consented to take Mr. Clifford Wigram's place, and that we shall be able to preserve the connection with the house which has from the first so efficiently aided us.

I am glad to say that during 1894, Guy's Hospital completed a most liberal scheme for the nurses of their private staff. The principle of it is, that, at the end of every financial year, the treasurer applies out of the profits such sum as he thinks fit for the purpose of purchasing paid-up polices in our Pension Fund for the nurses who have been in the service of the institution for a certain number of years. The whole of the money so appropriated is divided into a certain number of equal shares, and according to the length of service, the nurse receives a larger or smaller number of these shares. As regards the sisters and nurses in the hospital itself, Guy's Hospital follows a different plan to the majority of our federating institutions, for while other institutions require that the nurse shall take out a policy equivalent to the one for which the hospital pays, at Guy's the hospital pays for a policy of £11 5s., commencing at the age of fifty, against the nurse's contribution for a policy of £7 10s. only at the same age.

With regard to our finances, I would call your attention to the fact that there has been an increase of about £34,000 in the invested moneys, and I am glad to be able to repeat what I said last year—that, notwithstanding the extremely hard and difficult times through which we have passed, the value of our investments is in excess of the cost, and that there is no interest in arrear. The investments are very carefully selected by a sub-committee, which reports to a Finance Committee, and on their approval the whole subject is referred to the General Committee, and it is only after authority has been obtained from these three that any investment is made. We think, therefore, that every safeguard has been thrown round the care of your money, and our experience hitherto has shown that we have been very successful in avoiding any serious mistakes. The average rate of interest obtained is slightly over 4 per cent., and as your actuarial calculations are based upon 3 per cent., there is a margin of 1 per cent. profit on the moneys invested, which goes to accumulate towards the quinquennial bonus distribution.

The portion of our business which has given the Council the greatest anxiety has been the working of the sickness branch, as there were no data in existence when we started to accurately calculate the rate of sickness in a class which was specially exposed to contagion. I am glad to say that we are gradually building up an experience of our own, and it is satisfactory to observe that the rates between the premiums received and the sick pay has up to now shown a slight margin in our favour, and that the Sickness Fund consequently is steadily increasing. We have paid away in sickness allowances about £739, or £163 more than in 1893, the larger number of claims during the early part of the year being greater than the falling off during the last four months, when the sick pay was very much lower than during the corresponding months of the previous year. One hundred and forty-nine nurses have received sick pay, as against 129 the year before; and, including all cases, the average payment was £4 19s. 2d. per head.

The 144 policies discontinued during the year were held by 139 nurses. As seven were substituted policies, the number of nurses retiring or dying was 132. Of these, 31 gave up on account of marriage; 29 because they were giving up nursing; 11 died; 30 were unable to keep up their payments; and from various causes, 31. Of course the three first classes, covering 70 nurses, have withdrawn for such sound reasons that they were unavoidable. With regard to the remainder, especially those that were unable to keep up payment, I think it would be well if I suggested to the nurses that when they find themselves in such a position, it would be better for them to apply for the assistance of the Benevolent Fund, and thereby keep up their premiums for a time by a loan rather than to withdraw altogether, as experience shows that very many of them, after surrendering their policies, apply for re-insurance, and of course they come in at a worse rate from their increased age than if they had remained upon the original policy and kept it up. (Hear, hear.)

I am glad to say that in most instances the letters received from nurses on withdrawal testify to the fact that, had it not been for the Fund, they would not have saved anything, and they speak in terms of gratefulness of the assistance which the Fund has been to them.

Our profit in interest, that is to say, the interest received over and above the 3 per cent. required for actuarial purposes for the year, amounts to £1,500. Last year this profit was just over £1,000, and from the establishment of the Fund to December 31st, 1892, £702. You will, therefore, see that there is a steady and very satisfactory growth in our accumulations. (Hear, hear.)

The Benevolent Fund has been very active during the past year, having given in grants £465 out of their income, against £375 in 1893. Though the difference may seem very slight, it has, together with the changes of residence, added considerably to the office work, and I must take this opportunity of paying a well-merited tribute to the able and efficient manner in which our honorary secretary, Miss Pritchard, carries on her work, which necessitates a vast amount of correspondence, and careful inquiry in each case.

It is my pleasing duty to place on record the very satisfactory and willing manner in which our secretary, Mr. Dick, and the staff of the office have done their entire work. We have lost the services of a very faithful clerk, Mr Pocock, who left the service of the Fund in order to take a situation in which he gained an increased pay beyond that which it was in the power of the Fund to offer. We regret losing so faithful an employé and one who has always given satisfaction to the Council in the discharge o his duties.

The actual physical labour of the office is very great. The number of letters received during the year exceeded 16,000, and over 17,000 were dispatched from the office, making a total of 33,000 letters against about 30,000 last year. I only mention this, of course, to give an idea of the amount of work, so that members of the Fund may judge as to the moderation of the expenses with which the office work is conducted. As a further illustration, I might mention that with regard to the pension and sickness insurance premiums, the amount—about £34,000—was received in over 12,000 different remittances, each one of which requires, at least, three entries in the books. When to this we add entries of premiums in advance, surrenders, annuities paid, &c., the book entries are little short of 50,000 during the year.

Many nurses visit the office daily, over 1,000 having called during 1894. I think under the circumstances our work has been well done, and that we may justly be proud of the results obtained with so small a staff. With invested funds amounting to £200,000, a net annual income of over £40,000, a business steadily increasing, viewing the originality of the object and its peculiar constituency, the Fund is now in a position which none of its most sanguine promoters could at the outset have anticipated. (Applause.)

### The Ex-President of the Royal College of Surgeons.

Mr. Thomas Bryant, F.R.C.S.: I have great pleasure in seconding this report. Not one of us can doubt the success of this great Fund, and it certainly is a most gratifying thing to me to witness such a success, seeing that I have been connected with it since its establishment, and have carefully watched its progress. (Hear, hear.) I must confess that at first I was a little sceptical as to its success, but it is most gratifying to find that among such a large class of women we should have such a good spirit of economy, and such demonstrations of their intention to make provision for a later period of life. (Applause.) I am glad to say that I have been most agreeably disappointed in my first anticipations, and that the nurses have come to us literally in shoals, and in increasing numbers year by year. From a hospital point of view I feel more than gratified, for it has already led some hospital authorities, and will probably lead others, to join the Fund and make good provision for their nurses. (Hear, hear.) It is no secret that in years past the hospitals, with some exceptions, did not see the necessity of making provision for those who worked for them, and gave them the best years of their lives. It is, therefore, a most gratifying thing to find our hospitals, and more particularly the old hospital fund, Guy's, associating itself with this Fund. I should just like to point out to other hospitals the valuable scheme adopted at Guy's, because I should not like to see any fund separate from this, for we must all pull together, and pull together strongly, in order to make the matter a success; though certainly the scheme that has been laid down and adopted at Guy's seems in every way to be such a satisfactory arrangement as such an institution should have, and, in fact, any nursing institution; and it would be well to see that spirit further supported. I am quite satisfied to rise and support this very admirable and satisfactory report. (Applause.)

The report, accounts, and balance-sheet were unanimously adopted.

### Mrs. Burns Elected a Patroness.

Mr. Arthur H. A. Morton, L.C.C.: I beg to propose the election of Mrs. W. H. Burns as a patroness of the Fund. As Mr. W. H. Burns, one of the oldest of those connected with the Fund, is our chairman to-day, I am precluded from making many observations as to the way in which he has attended to its interests, but we all know that the Fund is very near and very dear to Mrs. Burns' heart. (Applause.) In view of her munificent contributions to the Junius S. Morgan Benevolent Fund, there is no position which she could more fittingly fill than that of one of the patronesses of the Fund.

Mr. G. Norman seconded the motion, and it was unanimously carried.

The Chairman: On behalf of my wife I most heartily thank Mr. Morton for the way in which he has spoken of her. I can assure you that both my wife and myself are glad that we should be associated together in such an excellent work. (Applause.)

### Election of Members of Council.

Mr. A. H. A. Morton then moved that the retiring members of the Council be re-elected. They were Mr. W. H. Burns, Mr. E. Rawlings, Mr. A. C. de Rothschild, Mr. T. Bryant, F.R.C.S., and Sir W. H. Broadbent, Bart., M.D. He said: Our chairman, who stands at the head of the list, has, perhaps, done more towards the establishment of this Fund than anyone present, and therefore I have the greatest pleasure in proposing that he and the other gentlemen named be re-elected.

Dr. E. C. Perry seconded the motion, and it was unanimously carried.

### The President and Thrift.

Mr. Burdett: As I was entering the room to-day I received a message from Sandringham, from our president, the Princess of Wales, to the effect that she is very carefully considering how she can emphasize her connection with the Fund and identify herself if possible, with every individual nurse. (Applause.) She regards every nurse connected with this Fund as one of her own nurses and I think she has hit upon a scheme which will have that effect, and which will make our Pension Fund known in every institution in this country and in the Colonies too. I think such a step will be of enormous advantage to the Fund. (Applause). Speaking on behalf of my colleagues and myself, and especially the chairman, we know that every additional nurse means an addition to our responsibilities. Our responsibilities have become somewhat large since the funds now exceed £200,000. Still we exist to help the nurses everywhere, and I believe the mere fact of nurses realizing that they have in their midst, in a great institution out of 90 nurses, six, say, who have found that by being members of the Pension Fund they are enabled, when attacked by sickness, to be absolutely independent of outside help, will stir up many to seek similar security for themselves. At the present time there are a great many nurses who think it is impossible to save enough money by regular monthly, or quarterly, or annual instalments to enable them to be safe against whatever may happen. But here on the books of the Pension Fund we have a number of nurses, approaching 4,000, who have proved the contrary. Every year this Fund shows that our Institution is becoming better understood by the nurses, and that saving is becoming fashionable and popular, because our contracts show an upward tendency. (Applause.) The nurses who join us now join for larger sums than formerly, and as all of us know, especially those who have much to do with nurses, there has been little or no increase recently in the wages paid, and therefore the increased premiums paid must represent the increased self-denial on the part of the nurses and the impression which they have that it is a desirable and a good thing to be independent, and independent by their own efforts. Therefore I hope the Press will let everyone know that so many nurses have had the courage to act as pioneers in this matter of saving to secure self-defence and protection for women workers, and that that fact will have an enormously good effect upon our institution and will immeasurably increase the numbers of those who will want to join the Fund. (Hear, hear.) We have always taken up the position, and shall continue to take it, that it is no part of our business to urge the nurses to join the Pension Fund. All that we do is to take care that every committee and matron in the country and the colonies has a copy of the report each year, as an intimation of what we are doing, and it is for them and their employés to decide for themselves whether or not they will elect to secure the comfort which we have to offer in the shape of permanent provision against sickness and old age. It was thought six years ago that it was an absolute impossibility for women workers of any class, but especially for nurses earning wages of £20 to £25 a year, to come in and save money enough to make themselves safe against the misfortunes of this life. But what has been the result? Every year our premium income has gone up, and now we have a premium income of something like £35,000, every penny of which comes from nurses' savings. That fact should give courage to women workers and all who have to do with women workers of all classes in this and other countries. (Applause.) There has been nothing like this Fund elsewhere in the world. It shows that women, like men, if they understand matters, and if only they are provided with reasonable security and opportunities, will come forward and defend and protect themselves adequately, as our nurses have done in this country and in the colonies.

### The Junius S. Morgan Benevolent Fund.

As I said last year, this Fund owed its existence in the first place to the late Mr. Junius S. Morgan, without whom there never would have been a Fund, and to the fact that the nurses were so grateful to him that they spontaneously raised the wonderful sum of nearly £2,300 in six weeks, to commemorate the memory of a man whom they had not known, but whom they appreciated and respected for holding out a strong hand to help them to unite solidly for self-preservation against any adversity that the world might have in store for them in the future. (Applause.) The fact of those nurses coming forward in that way re-acted on Mr. Morgan's family, who have since given very largely to the Fund in connection with its Benevolent Branch. You all know that if there was nothing else done but the work of the Benevolent Fund, it would be a glorious institution, seeing the good which it does for many self-respecting workers who, from the arduous character of their calling, are often stricken down in ways different from ordinary sickness. The Fund has in that way done an enormous amount of good, which it would, indeed, be difficult to exaggerate. (Applause.) This Benevolent Branch does two things. It enables us to see to a nurse who is taken ill, but who is not sufficiently ill to come upon our sick fund, because, to secure its benefits, nurses must be permanently disabled. Lady Rothschild and her committee enable us to take her, to have her examined, and to see exactly what she wants; to afford her the maximum opportunity of being able to resume her duties and her work. Many nurses get valuable advice; they are treated, and in many cases restored to sound health. But we have done more. We are able in the case of nurses who have joined the Fund—and we cannot much longer continue to help nurses, not members of the Fund, out of the Benevolent Fund, for we have not the funds to do it with—to give life pensions. We have now on this Fund at the present time twenty-five nurses who are life pensioners. These nurses are, most of them, amongst those who joined the Fund at the first. Perhaps they were forty-eight years of age, and they had therefore no prospect of being able to pay the premiums necessary to obtain for themselves an adequate pension. But as the nurses joined the Fund, and did what they could to help themselves as long as they could work, we have given them a life pension, which in no case is less than 10s. a week, or £26 a year, and we know that in this way a great deal of good has been done.

Influenza Patients and Nurses.

I should now like to ask the public, at a time when the majority of people have had an unpleasant though somewhat short bout of sickness, to consider where they would have been, very many of them, if it had not been that they succeeded in getting nurses? I would ask these patients very earnestly, because they include the strong and the young as well as the old and the weak, if they have done anything in any practical way to show their gratitude? They know that the nurses go from house to house, from sick bed to sick bed, without having even the variety of a doctor, who only goes in and out for a short time. But nurses must be always with their patients day after day, and perhaps week after week. If the influenza epidemic results in inducing a tenth part of the people who have suffered and smarted from that most deadly foe to think over and look at this question, then I am confident that so far as the Benevolent Fund is concerned it will continue to grow. I do think that if the people who have been benefited by nurses would just think about this matter, and of what this Fund is doing, we should find that without personal sacrifice they and their friends might give handsome aid to our Benevolent Fund. We have also a Donation Bonus Fund—another matter altogether. Nurses being working-women, I consider our first duty is to help them to help themselves; but I ask wealthy people if they have funds at their disposal to remember the Donation Bonus Fund. It stands at upwards of £30,000 to-day, but seeing that its income must go in bonuses to about three thousand nurses, and that we have between three and four thousand on our books, another £10,000 would be welcome, in order to give the nurses proper provision when they retire from their work. I should like to say that although we have received £40,000, I don't think forty thousand shillings, or even forty thousand pence, has come from the West-end of London. I think if the rich people there who have been clamouring and waiting of late, not by deputy but in person, at our hospitals and nursing institutions, begging and praying for nurses, would think of these things, our secretary would receive cheques from them. There must be some of the many thousands who have received relief during the influenza who would be glad to acknowledge the debt they owe by helping the nurses to help themselves. (Applause.)

Mr. King then handed in the scrutineers' report of the result of the ballot for seven representatives of annuitants and policy-holders which was announced as follows: "We have to report that 1,195 cards were returned, of which 54 were unsigned, and three contained more than seven names, leaving 1,138 policy-holders voting, with the following result: Miss L. M. Gordon, 1,129 votes; Miss E. Vincent, 1,102; Miss E. Fisher, 1,100; Miss K. H. Monk, 1,095; Miss F. C. Nott Bower, 1,088; Miss C. Davidson, 1,088. The next lady receiving the largest number of votes, namely, Miss Mary L. E. Dunn, superintendent of the Irish branch of the Queen Victoria Jubilee Institute, was also elected a representative, in accordance with the notice sent out by the Council. Forty-two other ladies were also voted for, the highest number received by any other one was five, and thirty only received one vote."

The Chairman thanked the scrutineers for their labours, and on his motion it was ordered that the ballot papers should be destroyed.

Mr. Burdett: I beg to be allowed to propose a vote of thanks to Mr. W. H. Burns, not only for his conduct in the chair, but also for the manner in which he has discharged his duties throughout the year as chairman of the Council. You, of course, do not know so well as we do the work which Mr. Burns does actually for the Fund. He is essentially a working chairman, and as a worker I do not know his equal. (Hear, hear). If there is one thing in London most difficult to get to-day it is a gentleman who will give his time and personal service in aid of any philanthropic movement of any kind whatever. I think I am correct in stating that Mr. Burns shares with me a sort of personal sentiment about this Fund, and that we both consider it a great privilege to be connected with it, and certainly, whether that be his reason or not, I do know that he has given an enormous amount of time and thought and attention to its interests. That has filled me not only with satisfaction, but with a kind of envy, for after all I cannot help regarding the Pension Fund as a kind of child of my own. However, we are all most grateful to him for the most valuable help he has given us, and we thank him from our hearts. (Applause.)

Mr. H. C. Gibbs seconded the motion, and it was also carried unanimously.

The Chairman: I am exceedingly grateful to Mr. Burdett and to Mr. Gibbs for the kind way in which they have placed this proposition before the meeting. I have done no more than my duty, and it has been a great pleasure to me to do it. I will not detain you with a long speech, but I can assure you that any length of words would be in inverse ratio to the gratitude I feel. (Applause.)

The proceedings of the annual meeting then concluded.

### Notes and Queries.

### Queries.

(97) *Burdett's Official Nursing Directory.*—Can you inform me if the age of nurses entering the Directory is to be recorded?—*Applicant.*

(98) *Nurses' Co-operation.*—Will you kindly give me the address of the Nurses' Co-operation in the Midlands?—*Nurse.*

(99) *Names.*—Why are some institutions called infirmaries and others hospitals? Are they all the same?—*A Sick Room Visitor.*

(100) *Baby.*—Will you tell me of some cheap books on bringing up children from the month? And also inform me whether an infant should be wakened for food in the day after being awake a great deal during the night?—*Nurse M.*

(101) *Sunderland.*—Can you give me the name of the Superintendent of District Nursing at Sunderland; also the secretary?—*Sister.*

### Answers.

(97) *Burdett's Official Nursing Directory (Applicant).*—The statement of age is not necessary.

(98) *Nurses' Co-operation (Nurse).*—The address of *The Nurses' Co-operation* is 8, New Cavendish Street, London. It has no town nor provincial branches.

(99) *Names. (A Sick Room Visitor).* In London and other large towns the title of "infirmary" is generally conferred solely on institutions under the Poor Law, namely workhouse infirmaries. In the country as well as in Scotland the county or general hospitals are frequently called infirmaries.

(100) *Baby (Nurse M.)*—"How to Feed the Baby," price 6d., published by the Ladies' Sanitary Association, Berners Street, is good, also "How to Nurse in our Own Homes," price 3d., published by Messrs. Wells Gardner, Darton, and Co., 44, Victoria Street. There are many other very good books rather more expensive. If the infant lies awake at night it cannot be well; you should pay special attention to its food. It is always a pity to waken a healthy child before he has had his sleep out; but a delicate baby may need to be sometimes roused to take nourishment. You should ask a doctor's advice. Often insufficient or too heavy bed-clothes will keep a child restless and wakeful. There were three articles in The Hospital last December, and a fourth on January 5th, entitled, "The Dietetic Management of Infancy." You should read them.

(101) *Sunderland (Sister).*—In "Burdett's Hospital Annual" you will find Miss Jefrey is the Matron of the Sunderland Nursing Institute, and Mr. W. H. Malling Hon. Secretary.

